# Supplementary material for: An Evaluation of Neuron-Specific Enolase as a Biomarker of Neurological Impact in Pacemaker-Implanted Patients with Atrial High-Rate Episodes: An Observational Study from Turkey
Source: Medicina (Kaunas). 2025 Feb 12;61(2):324. doi: 10.3390/medicina61020324 (PMC11857707; doi:10.3390/medicina61020324)
Supplement: Supplementary file 1 [file medicina-61-00324-s001.zip › medicina-3445310-supplementary.pdf]

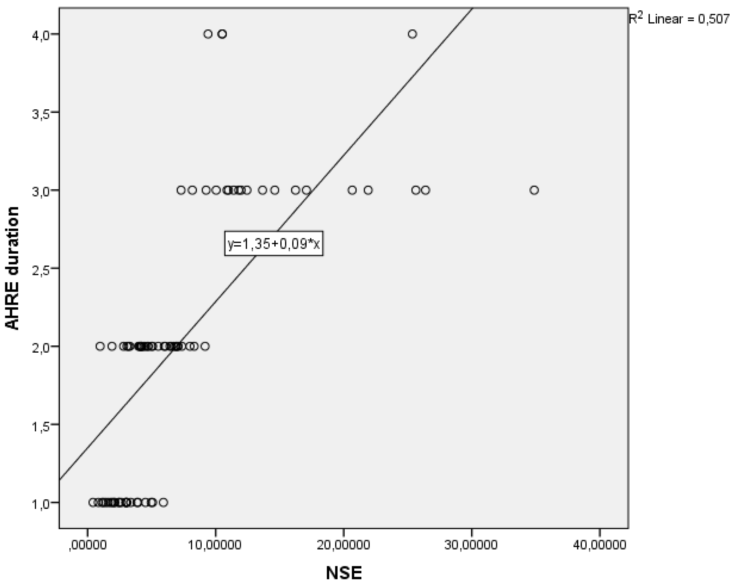

Figure S1. Correlation graph between Atrial High-Rate Episodes (AHRE) duration and Neuron-Specific Enolase (NSE) levels

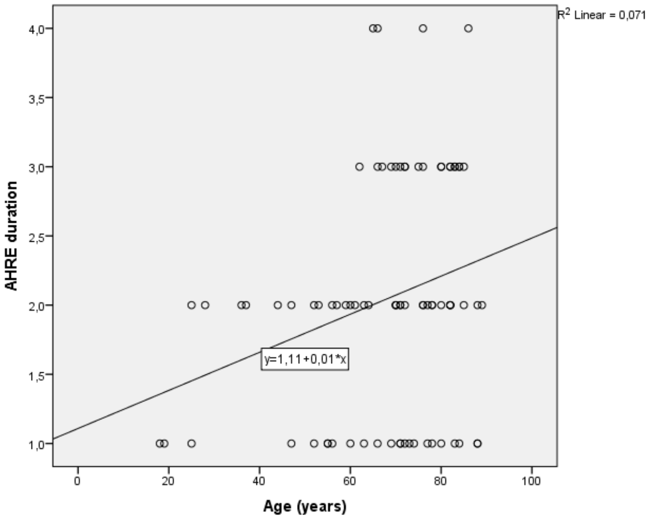

Figure S2. Correlation graph between Atrial High-Rate Episodes (AHRE) duration and age

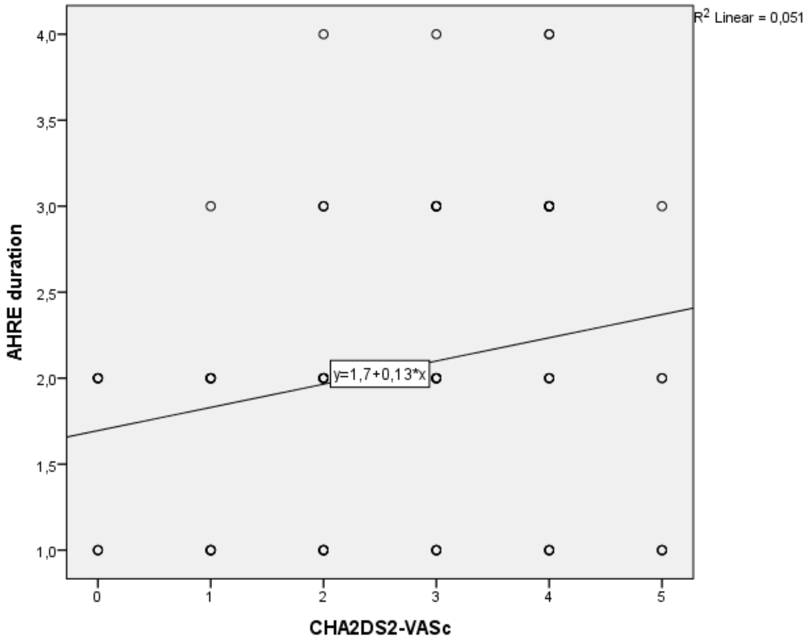

Figure S3. Correlation graph between Atrial High-Rate Episodes (AHRE) duration and virtual CHA_2DS_2-VASc score

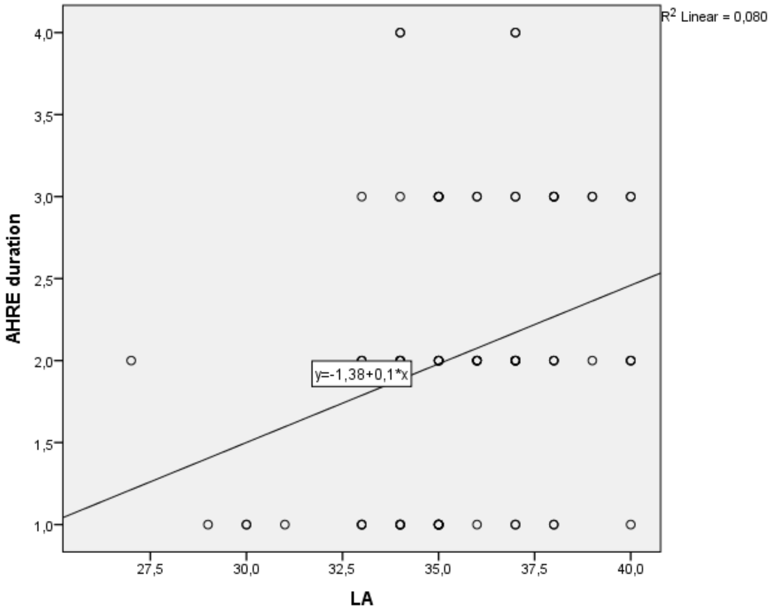

Figure S4. Correlation graph between Atrial High-Rate Episodes (AHRE) duration and Left Atrium (LA) size

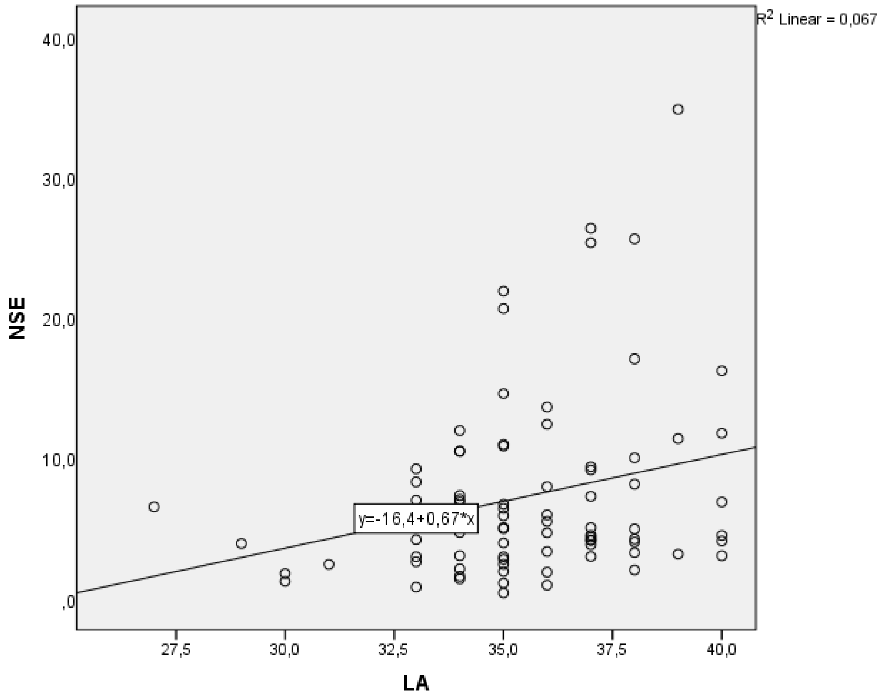

Figure S5. Correlation graph between Neuron-Specific Enolase (NSE) levels and Left Atrium (LA) size
